# MaskAppendix: Backbone-Enriched Mask R-CNN Based on Grad-CAM for Automatic Appendix Segmentation

**DOI:** 10.3390/diagnostics14212346

**Published:** 2024-10-22

**Authors:** Emre Dandıl, Betül Tiryaki Baştuğ, Mehmet Süleyman Yıldırım, Kadir Çorbacı, Gürkan Güneri

**Affiliations:** 1 Department of Computer Engineering, Faculty of Engineering, Bilecik Seyh Edebali University, 11230 Bilecik, Türkiye; 2 Radiology Department, Faculty of Medicine, Bilecik Şeyh Edebali University, 11230 Bilecik, Türkiye; betultryak@yahoo.com; 3 Department of Söğüt Vocational School, Computer Technology, Bilecik Şeyh Edebali University, Söğüt, 11600 Bilecik, Türkiye; mehmets.yildirim@bilecik.edu.tr; 4 General Surgery Department, Bilecik Osmaneli Mustafa Selahattin Çetintaş Hospital, 11500 Bilecik, Türkiye; dr.kadircorbaci@gmail.com; 5 General Surgery Department, Faculty of Medicine, Bilecik Şeyh Edebali University, 11230 Bilecik, Türkiye; gurkanguneri@gmail.com

**Keywords:** appendix segmentation, deep learning, CT imaging, mask R-CNN, grad-CAM, Detectron

## Abstract

Background: A leading cause of emergency abdominal surgery, appendicitis is a common condition affecting millions of people worldwide. Automatic and accurate segmentation of the appendix from medical imaging is a challenging task, due to its small size, variability in shape, and proximity to other anatomical structures. Methods: In this study, we propose a backbone-enriched Mask R-CNN architecture (MaskAppendix) on the Detectron platform, enhanced with Gradient-weighted Class Activation Mapping (Grad-CAM), for precise appendix segmentation on computed tomography (CT) scans. In the proposed MaskAppendix deep learning model, ResNet101 network is used as the backbone. By integrating Grad-CAM into the MaskAppendix network, our model improves feature localization, allowing it to better capture subtle variations in appendix morphology. Results: We conduct extensive experiments on a dataset of abdominal CT scans, demonstrating that our method achieves state-of-the-art performance in appendix segmentation, outperforming traditional segmentation techniques in terms of both accuracy and robustness. In the automatic segmentation of the appendix region in CT slices, a DSC score of 87.17% was achieved with the proposed approach, and the results obtained have the potential to improve clinical diagnostic accuracy. Conclusions: This framework provides an effective tool for aiding clinicians in the diagnosis of appendicitis and other related conditions, reducing the potential for diagnostic errors and enhancing clinical workflow efficiency.

## 1. Introduction

The appendix, a small, tube-like structure, extends from the cecum, the beginning of the large intestine, and is typically located in the lower right abdomen. Specifically, the appendix is found in the right iliac fossa, a region in the lower right part of the abdomen. Anatomically, it is a blind-ended pouch with a length that varies significantly among individuals, ranging from 5 to 35 cm [1]. As in the structure of the intestinal wall of the colon, the appendix’s wall histologically consists of mucosa, submucosa, muscularis externa, and serosa [2]. Physiologically, the appendix is thought to have a role in maintaining gut flora and responding to infections. However, its precise function remains debated, with theories ranging from a vestigial organ to a site for immune system regulation [3].

Appendicitis is an inflammation of the appendix [4]. It is a common surgical emergency characterized by acute or chronic inflammation, often leading to severe abdominal pain and, if untreated, potentially serious complications. The most common cause of abdominal pain is acute appendicitis [5]. Acute appendicitis is an important health problem that affects approximately 17 million people worldwide every year [6]. The most common cause of appendicitis is obstruction of the appendiceal lumen, which can be due to fecaliths (hardened stool), lymphoid hyperplasia, or foreign bodies. This obstruction impairs the drainage of mucus and other secretions, leading to increased intraluminal pressure, reduced blood flow, and subsequent bacterial overgrowth and inflammation [7,8,9,10,11]. Symptoms typically include right lower abdominal pain, nausea, vomiting, and fever. The condition can progress rapidly, and if the appendix becomes necrotic or ruptures, it can lead to peritonitis, a widespread abdominal infection, or abscess formation [12,13].

Treatment for appendicitis usually involves surgical removal of the appendix, known as an appendectomy, which can be performed via open surgery or laparoscopic technique [14]. A meta-analysis showed that appendectomy was significantly more effective in the treatment of patients diagnosed with acute appendicitis than in patients who received antibiotics alone [15]. This situation has shown us once again the importance of making the correct diagnosis of acute appendicitis. Diagnosis of acute appendicitis is difficult in emergency departments because diagnosis based solely on clinical evaluation has low sensitivity and low specificity [16]. In a study of 800,000 patients, in-hospital mortality was found to be 0.12% [17]. In some perioperative cases, appendectomy alone cannot be performed or is not sufficient. Complicated surgical procedures such as ileocecal resection, right hemicolectomy, and end ileostomy increase morbidity [18]. On the other hand, misdiagnosis of appendicitis may result in negative appendectomies. A negative appendectomy can cause long-term problems in addition to the risks of anesthesia and surgery. The increased incidence of inflammatory bowel disease and clostridium difficile infection in patients who have had appendectomy suggests that the appendix plays an important immunologic role [19]. Therefore, correctly identifying acute appendicitis and correctly grading it radiologically can allow the surgeon to prepare for possible morbidities and provide the opportunity to reduce morbidity. Early diagnosis and intervention are crucial to prevent complications and ensure favorable outcomes.

Computed tomography (CT) is a pivotal imaging modality in the assessment of appendiceal diseases, providing high-resolution cross-sectional images that aid in both diagnosis and management. Diagnosis of appendicitis is primarily clinical but can be supported by imaging such as ultrasound or CT to confirm the diagnosis and assess for complications [20,21,22]. When performed with oral or intravenous contrast, CT enhances the visualization of the appendix and surrounding structures, allowing for accurate differentiation between appendicitis and other abdominal pathologies. Considering the sensitivity and specificity of CT, it is a very successful radiological examination method in the diagnosis and management of acute appendicitis. However, the urgency of the disease increases the workload of radiologists in the evaluation of these CTs and causes loss of time when planning treatment for patients [23]. Therefore, the development of technologies that interpret CTs at least as accurately as radiologists and faster than them is an important need for clinicians. A better segmentation of the appendix significantly improves diagnostic accuracy, accelerates decision making and allows for more effective treatment planning. Precise segmentation may allow clinicians to visualize the appendix more clearly on CT scans, making it easier to identify signs of inflammation, perforation, or abscess development. By accurately delineating the appendix from surrounding structures, the risk of missing important diagnostic details can be reduced. In emergency situations, faster and more reliable identification of appendicitis can be life-saving. Automated segmentation of the appendix simplifies the radiology workflow by providing instant and accurate contours of the appendix, allowing radiologists to make faster and more assured decisions [24].

Because the appendix is a small, finger-shaped organ in the lower right abdomen, it is sometimes difficult to see, especially on medical scans. The appendix usually causes no symptoms or discomfort in everyday life. However, if appendicitis develops as a result of inflammation or infection of the appendix, the appendix must be removed urgently as it can lead to serious complications. In addition, the appendix can be located in many different places in the abdominal cavity [25]. This variability makes the diagnosis difficult, as the inflammation can be different depending on the location. On the other hand, misdiagnosing appendicitis as another condition can delay necessary surgery and increase the risk of complications such as perforation and peritonitis [26]. Well-defined segmentation delineates the appendix boundaries, ensuring radiologists can confidently identify the appendix. It is therefore very important to segment the appendix accurately with precise boundaries. Previous studies on appendix segmentation are scarce or very limited. In particular, it can be seen that the proposed studies on the appendix mostly focus on the classification of normal appendix and appendicitis and the prediction of appendicitis. In one of these studies, Marcinkevics et al. [27] applied prediction of appendicitis in children based on comprehensive medical history, clinical assessment, laboratory parameters, and abdominal ultrasound. The study used machine learning approaches including logistic regression, random forests and boosting machines. In another study proposed by Byun et al. [28], CT image review, laboratory tests, and clinical features were used with a decision tree-based machine learning approach to predict appendicitis in children.

In medical imaging and image processing, artificial intelligence (AI) is emerging as a leading field [29]. AI is a powerful tool for the transformation of raw data into meaningful insights [30]. The emergence of deep learning has transformed the field of AI and has significantly changed the field of data analytics [31]. Significant contributions have been provided to improve clinical decision support systems by integrating deep learning into clinical applications [32]. In particular, the quality of image segmentation has been significantly improved through the use of deep learning methods [33]. Deep learning approaches also provide significant benefits to medical image segmentation, particularly in terms of feature learning, automatization, and improved accuracy. Deep learning-based methods automatically learn structured features from image data, detecting both low-level features such as edges and textures and high-level features such as shape and structure, without the need for manual feature extraction [34]. Deep learning approaches have achieved state-of-the-art performance in medical image segmentation tasks [35]. In recent years, many deep learning-based methods have been proposed in evaluation of appendiceal diseases. These computer-aided methods have enhanced diagnostic accuracy, streamlined workflows, and offered novel insights. In the context of appendiceal diseases, deep learning models can assist in detecting appendicitis, identifying appendiceal abnormalities, and predicting disease progression. For instance, these models can be trained to segment the appendix accurately and to classify images based on the presence of inflammation, perforation, or other complications. In addition, deep learning-based systems can assist radiologists by providing additional diagnostic insights and reducing the burden of image analysis [36]. In one of the proposed studies based on deep learning, using a reinforcement learning based on CNN, Al et al. [37] proposed an approach that can detect acute appendicitis in abdominal CT images. Lee et al. [38] focused on the distinction between acute appendicitis and diverticulitis using 2D CNN in abdominal CT scans. Liang et al. [39] presented an approach using combined deep learning and radiomics to differentiate between complicated and uncomplicated cases of acute appendicitis. Park et al. [40] proposed a CNN method for the classification of appendicitis using CT scans of patients with abdominal pain presenting to the emergency department. Rajpurkar et al. [41] presented a 3D deep learning model (AppendiXNet) for classification of appendicitis from CT scans. In another study, Park et al. [36] proposed the classification of normal appendix, diverticulitis, and acute appendicitis using EfficientNet, a deep learning model.

Previous studies on the appendix have mostly focused on predicting appendicitis using machine learning with different findings and classifying appendicitis from CT scans. Especially before the diagnosis of appendicitis, it is important to accurately segment the appendix organ with its real boundaries. At this stage, the appearance of borders can make it difficult to make a correct diagnosis or cause complications in the treatment process. Appendicitis is diagnosed using a combination of the doctor’s clinical experience, physical examination findings, imaging techniques, and laboratory tests. However, false positive or false negative results may be obtained due to the limitations mentioned above. Therefore, in patients with suspected appendicitis, the boundaries of the appendix must be precisely delineated. The main reason for our study is this need for automatic evaluation of appendix. Based on this, we aimed to develop a model that can evaluate the appendix on CT images. In this study, we propose the MaskAppendix for appendix segmentation, examining its performance in terms of accuracy, computational efficiency, and robustness in CT imaging modality. Furthermore, we compare the effectiveness of MaskAppendix with the state-of-the-art methods to highlight the strengths and limitations of each model. Our results demonstrate the potential of MaskAppendix to transform the landscape of appendix segmentation, paving the way for more streamlined and reliable diagnostic workflows in detection of appendix. Applying MaskAppendix to appendix segmentation leverages its ability to precisely delineate the appendix from surrounding structures in complex CT images. This capability is particularly valuable given the appendix’s small size and the variability in its anatomical location and appearance across different patients. By automating the segmentation process, MaskAppendix can potentially reduce the workload of radiologists and improve the consistency of appendix identification in clinical practice. The highlights of this study are:

- Automatic segmentation of the appendix is provided on CT scans;
- Segmentation of the appendix is applied with high performance using MaskAppendix on the Detectron platform;
- An appendix CT image dataset is created for this study;
- The precise appendix segmentation on CT scans with localization is enhanced using Grad-CAM;
- This framework provides an effective tool for aiding clinicians in the diagnosis of appendicitis;
- The MaskAppendix method achieves state-of-the-art performance in appendix segmentation.

The following sections of this study are organized as follows. In Section 2, the details of the appendix dataset prepared for this study are presented and the infrastructure of the proposed MaskAppendix is explained in detail. In Section 3, the results of the experimental studies are presented. An Ablation Study is also conducted in this section. The findings and evaluations are discussed in Section 4 and the results are compared with state-of-the-art methods. Section 5 contains conclusions and future work.

## 2. Materials and Methods

**Figure 1:**
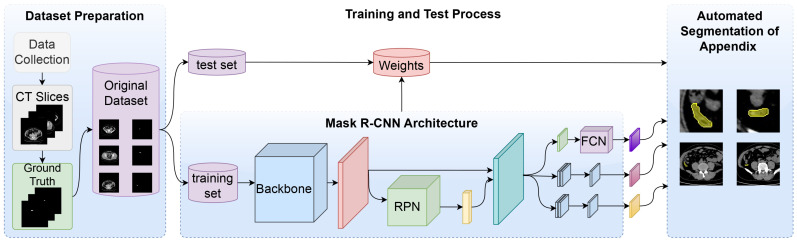
Overall architecture of the proposed MaskAppendix model for automatic segmentation of the appendix.

The general methodology of the MaskAppendix model proposed in this study for the automatic segmentation of the appendix is shown in Figure 1. In the methodology of MaskAppendix, in the first step, the dataset consisting of CT scans was prepared and divided into two categories as training and test data. In the second step, the MaskAppendix deep learning model was trained on the prepared training set and the optimal network weights were obtained. In the last step, the appendix was segmented on the test set using the best weights obtained in the training phase for the MaskAppendix model and the appendix segmentation results of the proposed model were evaluated. The model exploits the strengths of each component: Mask R-CNN’s segmentation precision, ResNet’s powerful feature extraction, Grad-CAM’s transparency and Detectron’s modularity. In the proposed MaskAppendix model, the ResNet50 and ResNet101 architectures serve as the backbone of Mask R-CNN, providing the deep and rich features that are critical for detecting and segmenting small and complex structures such as the appendix. By integrating Grad-CAM into this architecture, it is possible for clinicians to visualize which features or areas of the CT scan influenced the model’s decision. The combination of Mask R-CNN’s instance segmentation, ResNet’s deep feature extraction, and Grad-CAM’s explainability ensures that the model not only provides highly accurate segmentations, but also provides insight into how those decisions were achieved. In addition, Detectron simplifies the deployment of the Mask R-CNN model with a ResNet backbone. It enables efficient training, testing, and fine-tuning of the segmentation models, saving time and computing resources.

### 2.1. Dataset

In this study, an original dataset of CT scans was created for automatic segmentation of appendix. Abdominal CT images of different patients in the dataset were collected at Bilecik Training and Research Hospital in the presence of physicians from the Radiology and General Surgery Departments. The abdominal CT scans were obtained from 235 patients who presented to the emergency department with abdominal pain and suspected acute appendicitis. A total of 153 of the patients were male and the remaining 82 were female, aged between 18 and 91 years. Patients were evaluated in the ward in terms of demographic and laboratory characteristics as well as the presence or absence of appendicitis. Therefore, 129 of the patients were diagnosed with acute appendicitis by physicians and the remaining 106 were healthy with a normal appendix. Patients were placed in the supine position during the CT scan, and slices were acquired from the upper abdomen to the lower abdomen. CT scans were performed between March 2022 and April 2024. The CT scanner and some important acquisition parameters are listed in Table 1. The study was ethically approved by Non-Interventional Clinical Research Ethics Committee of Bilecik Şeyh Edebali University according to decision number 13 of the 8th meeting on 5 December 2023. In addition, the patients whose data were included in the dataset created as part of the study agreed to the use of their data for research purposes.

**Figure 2:**
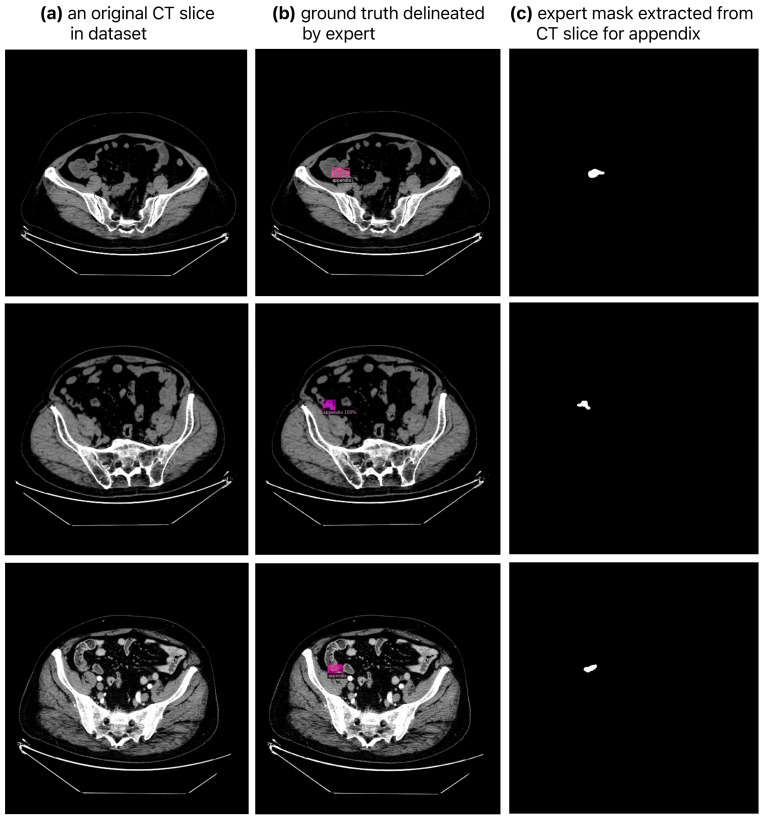
Original CT in dataset slices and the appendix region masks in these CT slices extracted by expert physicians.

In the dataset created for the appendix segmentation in the study, the patients’ CT scans were obtained in DICOM format. During data preparation, axial slices from each patient’s CT scan that were considered to contain the appendix were identified by specialized physicians. For the dataset, a total of 940 slices, 459 slices from scanned patients with appendicitis and 481 slices from healthy individuals, were identified as suitable for appendix segmentation. The appendix regions in these CT slices were extracted by expert physicians using the ITK-SNAP v4.0.2 software [42,43], as shown in Figure 2. For each original CT slice in the dataset shown in Figure 2a, the ground truth mask delineated by the expert is shown in Figure 2b and the appendix region extracted on the CT slice is shown in Figure 2c. In addition, the ground truth masks of the appendix regions for the dataset were saved in NIfTI format. Image pre-processing techniques were then used to enhance the CT images by histogram equalization, noise removal and line sharpening.

### 2.2. Proposed Mask R-CNN Model

**Figure 3:**
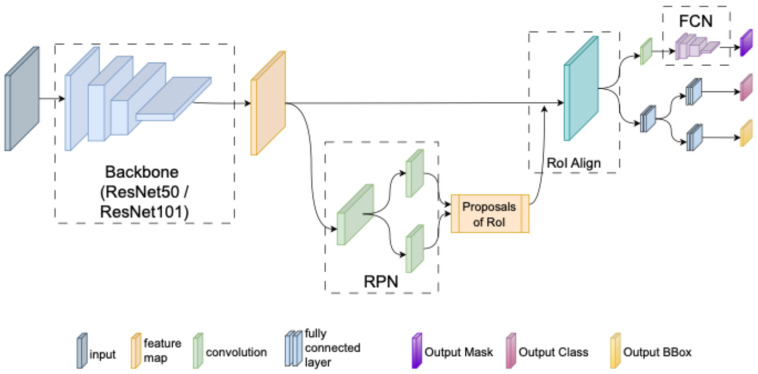
The proposed Mask R-CNN framework for automatic segmentation of the appendix on CT images.

Mask R-CNN is one of the current deep learning methods widely used for object recognition from images and accurate segmentation of objects in images [44]. Mask R-CNN is also a heuristic extension of faster R-CNN [45], another widely used method for object recognition, and has a very easy to train architecture. A typical Mask R-CNN architecture consists of three stages [46,47]. In the first stage, as in the faster R-CNN, the input image is processed by a backbone network and passed to a Region Proposal Network (RPN) to extract features and generate the feature map [47]. In the second stage of Mask R-CNN, RPN is applied to the associated candidate regions. Here, inspired by fast R-CNN [48], region of interest alignment (RoIAlign) is performed by applying a RoI pooling layer to the candidate bounding boxes to make each candidate region the same size. In the final stage of Mask R-CNN, the bounding boxes of the objects are classified by passing them through a Fully Connected Layer (FCL). Unlike Faster R-CNN, Mask R-CNN uses a Fully Convolutional Network (FCN) to create a mask in parallel with each bounding box [49]. In this study, a deep learning model based on the Mask R-CNN architecture shown in Figure 3 is proposed for appendix detection on axial CT scans.

In the masked R-CNN, the loss function given in Equation (1) is used for the mask part (head). In this equation, $L_{mask}$ calculates the pixel-wise cross-entropy between the target mask and the estimated mask. $L_{Mask\;R-CNN}$ denotes a multitasking loss function based on the loss function used for fast R-CNN [48,50]. Here, the classification loss $L_{class}$ and the bounding box loss ($L_{bbox}$) are the same as in fast R-CNN [48]. $L_{mask}$ is the average binary cross-entropy loss for the RoI associated with the ground truth class [49].

(1) $$L_{Mask\;R-CNN}=L_{class}+L_{bbox}+L_{mask}$$

### 2.3. ResNet

**Figure 4:**
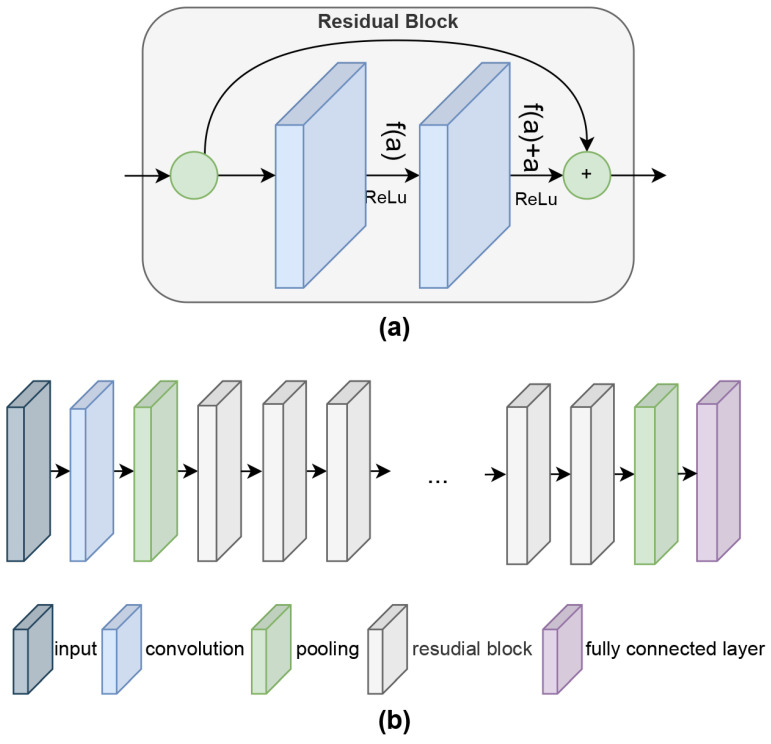
(**a**) Internal structure of the ResNet backbone network, (**b**) depth generation with residual block in ResNet50 and ResNet101.

In the Mask R-CNN architecture, the ResNet [51] architecture is widely used as the backbone to perform feature extraction from images differently. The architecture of the ResNet network, which is an improved combination of CNN structures, consists of block structures [52]. The vanishing gradient problem, which arises due to the large number of layers in deep learning models, is solved by using the residual blocks introduced by the ResNet network. Residual blocks allow an input to be directly summed with the output, creating short-cut connections. In the ResNet architecture, residual links can be created at different depths. In this study, ResNet50 and ResNet101 networks with a depth of 50 and 101 layers, respectively, are used as the backbone in the first stage of the Mask R-CNN architecture for feature extraction and feature map generation from images. Since the ResNet50 and ResNet101 architectures are deeper ResNet networks, bottleneck blocks are used in their infrastructure. In bottleneck blocks, 1 × 1 convolution is used to reduce the number of channels, 3 × 3 convolution and 1 × 1 convolution are used to restore the number of channels. In this way, high accuracy and speed benefits can be achieved in experimental studies. First, for each abdominal CT image, the features are extracted using the ResNet backbone and the window shifting method is used to determine the bounding box recommendations associated with the feature map. The internal structure of the ResNet backbone network is shown in Figure 4a. On the other hand, Figure 4b shows the depth generation with residual block in ResNet50 and ResNet101.

### 2.4. Detectron2 Platform

The Detectron infrastructure is a tool that provides advanced object recognition and segmentation applications, in particular the Mask R-CNN architecture. Detectron2 [53] is a state-of-the-art open-source library developed by Facebook AI Research and compiled entirely in PyTorch. Detectron2 also offers a dynamic architecture, faster training, and easier model configuration. In addition, the PyTorch-based Detectron2 infrastructure includes different configurations for Mask R-CNN and can be used directly with fine-tuned parameters. Because of these features, the MaskAppendix model proposed in this study was run on the Detectron2 platform.

## 3. Experimental Studies

In this study, several experimental analyses were performed to verify the performance of the proposed Mask R-CNN based model with two different backbones running on the Detectron2 library for appendix detection from CT images, and the results and findings were evaluated. All implementations of the proposed MaskAppendix were done using the Python programming language. A workstation computer, whose hardware components are listed in Table 2, was used for the experimental studies. Two GPUs were used to increase the processing speed and to obtain more effective segmentation results. The model used is based on the Mask R-CNN framework on the open source Detectron2 platform.

In this study, several improvements were performed for test evaluation and training using the model developed on the open-source Mask R-CNN framework on the Detectron2 platform. Hyperparameter selection and optimization are critical to the success of deep learning models such as MaskAppendix. In this study, by systematically experimenting with different hyperparameters, performing ablation studies, and using grid search methods, the best performing configuration was identified. This process ensures that the model achieves high segmentation accuracy, robustness, and computational efficiency, making it a valuable support for automatic appendix segmentation in CT scans. The optimization of the hyperparameters used for the MaskAppendix model proposed in this study for automatic appendix segmentation is presented in Table 3. These parameters have been identified with the best results based on the tests carried out in the experimental studies. ResNet50 or ResNet101 was used as the backbone for ablation in the Mask R-CNN network. In addition, it was noted that faster results were obtained using two GPUs on the computer where the experimental analysis was performed. On the other hand, the number of worker threads or processes used for data loading and the number of images processed in each training batch were 16 and 2, respectively. The learning rate in the network was chosen to be 0.001 for better learning of details. Furthermore, the optimal CHECKPOINT_PERIOD for training the network is 1000 and MAX_ITER is 100,000, which was also verified by the performance on the test set. For the sizes of the anchors for object detection, the values 16, 32, 64, 128, and 256 were applied as a list, and the number of RoI was set to 256.

In this study, the appendix segmentation performance of the proposed MaskAppendix architecture was evaluated using both distance-based and overlap-based metrics. For appendix segmentation from abdominal CT images, the segmentation results obtained using the proposed MaskAppendix model and the ground truth masks delineated by expert physicians were compared with the key metrics Dice Similarity Coefficient (DSC), Jaccard Similarity Index (JSI), Volumetric Overlap Error (VOE), Average Symmetric Surface Distance (ASD), and Hausdorff Distance 95 (HD95) to demonstrate the accuracy of the achieved segmentation. These metrics are given in Equation (2), Equation (3), Equation (4), Equation (5), and Equations (6), (7), and (8) respectively. In the equations given for the criteria used, the area of the ground truth masks delineated by the reference expert physicians is denoted as $A_{GT}$ and the area masked by the proposed model is denoted as $A_{MA}$. DSC calculates the overlap ratio of the areas of the ground truth masks delineated by the experts and the area masked by the proposed method [54]. On the other hand, by taking into account the intersection of the ground truth mask areas and the masked areas using the proposed method, the overlap ratio can be calculated from a different perspective with the JSI metric [55]. While the VOE key metric is used to determine the overlap error for the ground truth mask regions and the masked regions using the proposed method, the difference that physically occurs between the ground truth mask regions and the masked regions using the proposed method is calculated by ASD. In the calculation of the ASD metric, *d* represents the minimum distance between two segmentation masks. HD95 is used in the metric space to show how far apart the ground truth mask areas and the areas masked using the proposed method are from each other.

(2) $$DSC\left(A_{MA},A_{GT}\right)=\frac{2\left|A_{MA}\cap A_{GT}\right|}{\left|A_{MA}\right|\cup \left|A_{GT}\right|}\times \;100$$

(3) $$JSI\left(A_{MA},A_{GT}\right)=\frac{\left|A_{MA}\cap {\;A}_{GT}\right|}{\left|A_{MA}\right|+\left|{\;A}_{GT}\right|-\left|A_{MA}\cap {\;A}_{GT}\right|}\times \;100$$

(4) $$VOE\left(A_{MA},A_{GT}\right)=100\times \left(1-\frac{\left|A_{MA}\cap A_{GT}\right|}{\left|A_{MA}\cup A_{GT}\right|}\right)$$

(5) $$ASD\left(A_{MA},A_{GT}\right)=\frac{1}{\left|S(A_{MA})\right|+\left|S(A_{GT})\right|}\left(\begin{array}{c} \sum_{S_{A_{MA}}\in \;S\left(A_{MA}\right)}d\left(SA_{MA},\;S\left(A_{GT}\right)\right)+\\ \sum_{S_{A_{ref}}\in \;S(A_{GT})}d\left(SA_{GT},\;S(A_{MA})\right) \end{array}\right)$$

(6) $$hd(A_{MA},A_{GT})=\underset{\mathrm{x\epsilon}A_{\mathrm{MA}}}{\max}\underset{\mathrm{y\epsilon}A_{\mathrm{GT}}}{\min}{\left\|x-y\right\|}_{2}$$

(7) $$hd(A_{GT},\;A_{MA})=\underset{\mathrm{x\epsilon}A_{\mathrm{GT}}}{\max}\underset{\mathrm{y\epsilon}A_{\mathrm{MA}}}{\min}{\left\|x-y\right\|}_{2}$$

(8) $$HD95\left(A_{GT},\;A_{MA}\right)=max\;\left(hd(A_{MA},A_{GT}),hd(A_{GT},\;A_{MA}\right)$$

**Figure 5:**
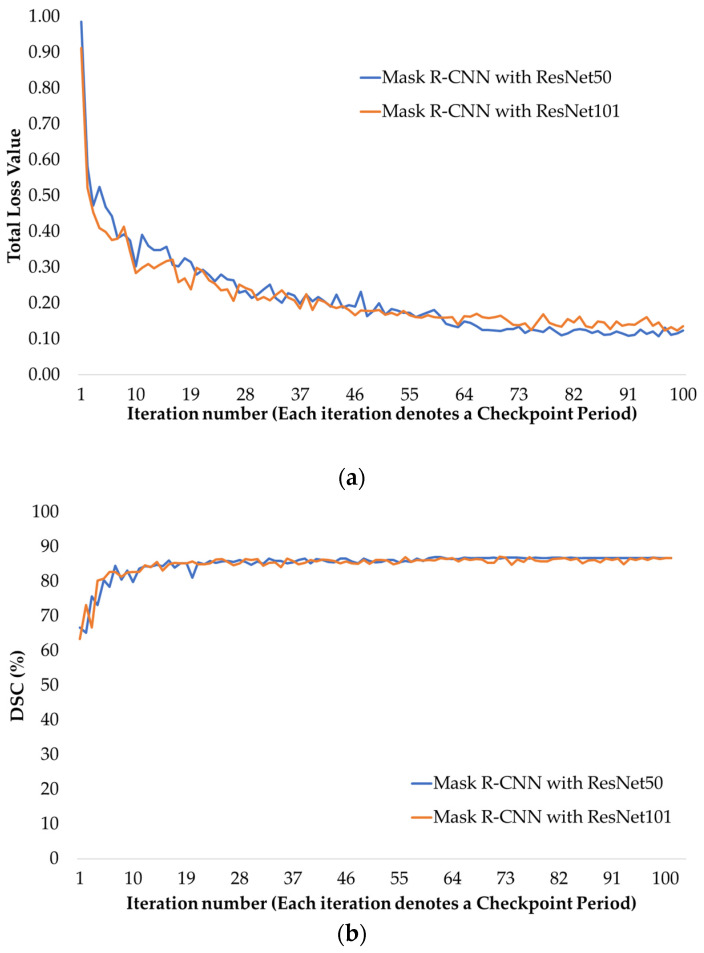
(**a**) Progression of total loss values during training using ResNet50 and ResNet101 backbone in Mask R-CNN network, (**b**) DSC progression using Mask R-CNN with ResNet50 and Mask R-CNN with ResNet101 on the test set.

In the dataset prepared within the scope of this study, suitable slices of the CT abdominal scans obtained from the patients, in which the appendix region is visible, were selected in the presence of expert physicians. The effectiveness of the proposed method was evaluated by means of various analyses, using the results of the experimental studies carried out on these slices for appendix segmentation. From a total of 940 CT images selected from appendicitis and normal patients, 752 (~80%) were used for the training phase of the MaskAppendix model and the remaining 188 (~20%) were used for the test phase. The training set contains 378 normal appendix images and 374 appendicitis images, while 103 of the test set are normal appendix images and the remaining 85 are appendicitis images. In addition, for the MaskAppendix model proposed in this study, a training phase was performed separately for ResNet50 and ResNet101 backbone networks on the training set. Time and performance evaluations of the training phase were performed. Figure 5a shows the total loss values obtained according to the number of iterations for each backbone in the training phase using ResNet50 and ResNet101 networks as the backbone in the Mask R-CNN architecture. Although the loss values obtained for the training phase of the Mask R-CNN network for both backbones were quite close to each other, a slightly lower loss value was achieved for the Mask R-CNN with ResNet101. On the other hand, in the test phase of the proposed MaskAppendix model, measurements were made separately for the check point in each period using the weights obtained in training. Figure 5b shows the progression of the DSC key metric as a function of the number of iterations for Mask R-CNN with ResNet50 and Mask R-CNN with Res-Net101 networks on the test set. For DSC, it can be said that the segmentation results for both networks are quite similar, and again a slightly higher DSC is obtained for Mask R-CNN with ResNet101. Here, each iteration number represents a checkpoint period.

### 3.1. Results

**Figure 6:**
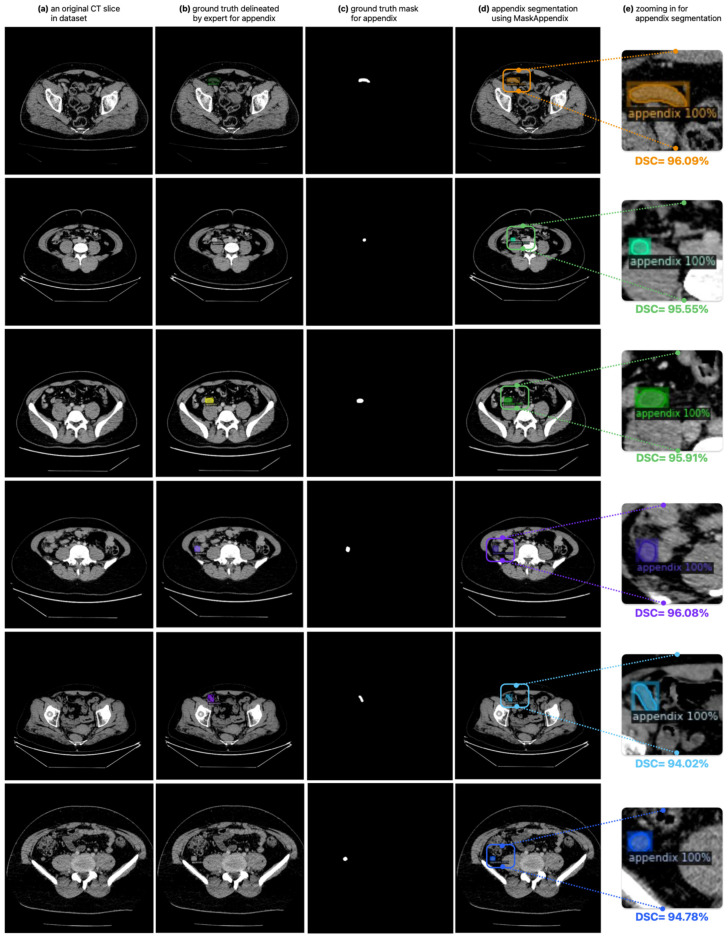
The successful automatic segmentation of the appendix in CT slices using the proposed MaskAppendix model.

In the experimental studies conducted with the proposed MaskAppendix model, automatic segmentation of the appendix from CT images was successfully performed, as shown in Figure 6. Although the appendix covers a small area on the abdominal CT, its pattern is similar to other tissues, and its boundaries are not clear in some CT slices, the proposed MaskAppendix model successfully masked the appendix. The ground truth delineated by the expert raters for the appendix in an original abdominal CT scan in the dataset in Figure 6a is shown in Figure 6b, and the ground truth only mask showing the appendix extracted from the whole CT slice is shown in Figure 6c. Figure 6d shows the automatic segmentation of the appendix in the CT image using the proposed MaskAppendix, while Figure 6e shows the zoomed segmented appendix region with the overlap ratio and the achieved DSC score. It can be seen that the segmentation results of the expert physician proposed MaskAppendix model with the delineated ground truth masks have very high overlap rates and the segmentation is successful with high DSC achieved.

In this study, the average results of the CT scans in the test set for the proposed MaskAppendix model on the dataset originally prepared for appendix segmentation are given in Table 4, based on the DSC, JSI, VOE, ASD, and HD95 key metrics. In addition, the segmentation results obtained with MaskAppendix for appendix segmentation are compared with the segmentation results obtained with the state-of-the-art methods U-Net [56] and DenseNet [57]. While U-Net is a deep learning architecture based on encoders and decoders widely used in medical image segmentation, DenseNet is a method based on connecting each layer in the network to the outputs of all previous layers. As can be seen from this table, the results of the DSC, JSI, VOE, ASD, and HD95 metrics used to measure segmentation performance in the appendix show that the proposed MaskAppendix model is more successful. In terms of the DSC key metric, 79.88%, 85.94%, and 87.17% were obtained for DenseNet, U-Net, and MaskAppendix (Mask R-CNN with ResNet101), respectively. Moreover, the proposed method is more successful for JSI, another key metric used for overlap accuracy, and VOE, a metric used for overlap error measurement. On the other hand, for ASD and HD95, key metrics calculated according to the smallest distance between two clusters, results with smaller distances than U-Net and DenseNet architectures were obtained using Mask R-CNN with ResNet101, 0.47 mm and 3.70 mm, respectively. The MaskAppendix model is more successful than U-Net and DenseNet in appendix segmentation due to its ability to perform instance segmentation with Mask R-CNN, enhanced feature localization with Grad-CAM, and deeper feature extraction with ResNet101. It is particularly well-suited to the small, variable and complex nature of the appendix due to its region proposal network, anchor boxes, and two-stage segmentation process, resulting in more accurate and reliable segmentation results.

### 3.2. Ablation Study

In this study, the results of the ablation experiments were also evaluated to verify the effect of the ResNet50 and ResNet101 modules on the segmentation performance of the MaskAppendix architecture, proposed as the backbone for the segmentation of the appendix region on CT slices. As shown in Table 5, the results obtained by optimizing the same network hyperparameters using ResNet50 and ResNet101 separately in the Mask-R-CNN architecture are compared for the key metrics DSC, JSI, VOE, ASD, and HD95. When ResNet101 is used as the backbone in the Mask R-CNN architecture (MaskAppendix), the appendix segmentation results are slightly better than Mask R-CNN with ResNet50. For the DSC and JSI metrics used for overlap accuracy, Mask R-CNN with Res-Net101 showed higher accuracy with values of 87.17% and 78.11%, respectively. In addition, for the VOE metric, which indicates the overlap error, Mask R-CNN with ResNet101 (MaskAppendix) has a lower error than Mask R-CNN with ResNet50. On the other hand, it can be said that Mask R-CNN with ResNet101 is more successful with a lower distance of 0.47 mm for the ASD metric used to evaluate the accuracy in the Appendix segmentation results, and Mask R-CNN with ResNet50 is more successful with 2.68 mm for the HD95 metric.

**Figure 7:**
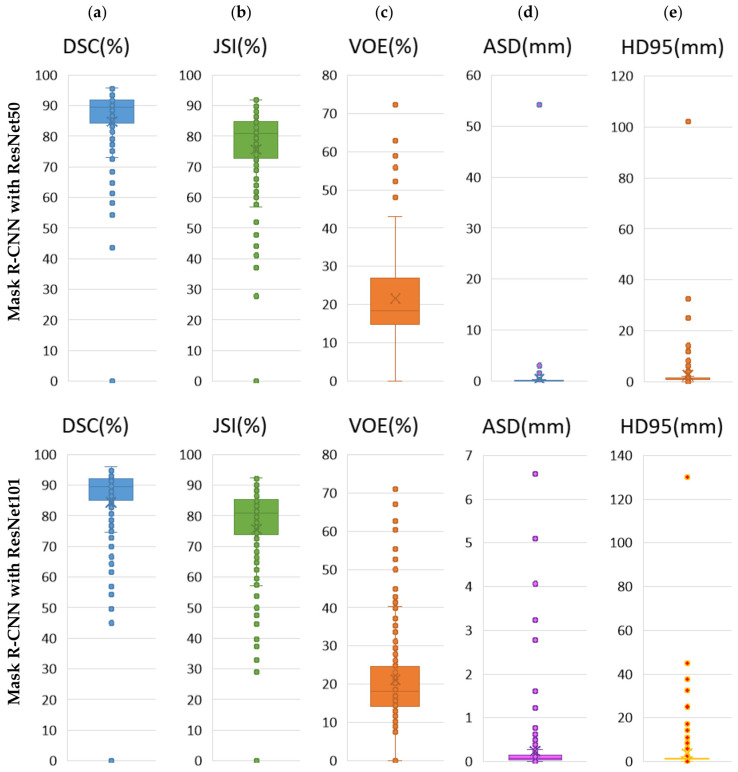
Boxplot evaluation of the results of ablation experiments carried out to verify the effect of ResNet50 and ResNet101 modules proposed as backbone in the Mask R-CNN architecture on segmentation performance. Boxplot for (**a**) DSC, (**b**) JSI, (**c**) VOE, (**d**) ASD, and (**e**) HD95.

The boxplot evaluation of the results of the ablation experiments performed to verify the effect of the ResNet50 and ResNet101 modules, proposed as the backbone in the mask R-CNN architecture, on the segmentation performance is presented in Figure 7. Figure 7a shows the box plots verifying the segmentation results of the DSC metric, Figure 7b the JSI metric, Figure 7c the VOE metric, Figure 7d the ASD metric, and Figure 7e the HD95 metric for the Mask R-CNN with ResNet50 and Mask R-CNN with ResNet101 models. It can be seen that the results of the boxplot averages for Mask R-CNN with ResNet50 and Mask R-CNN with ResNet101 for each metric are close to each other.

## 4. Discussion

To the best of our knowledge, the studies proposed for appendix segmentation on CT scans are very limited, if not non-existent. It seems that the studies proposed for appendix detection are mostly proposed for the classification of normal appendix and appendicitis [36,37,40,41]. Compared to the segmentations obtained with the state-of-the-art U-Net and DenseNet methods, in our study we achieved a segmentation performance for the appendix with 87.17% DSC using the proposed MaskAppendix deep learning model. On the other hand, in our previous study [24], the appendix was segmented with a DSC score of 85.94% using the U-Net architecture, while in this study a DSC score of 87.17% was achieved using MaskAppendix. Comparing the appendix segmentation results in this study with our previous study, we can see that the results for appendix segmentation are more successful.

**Figure 8:**
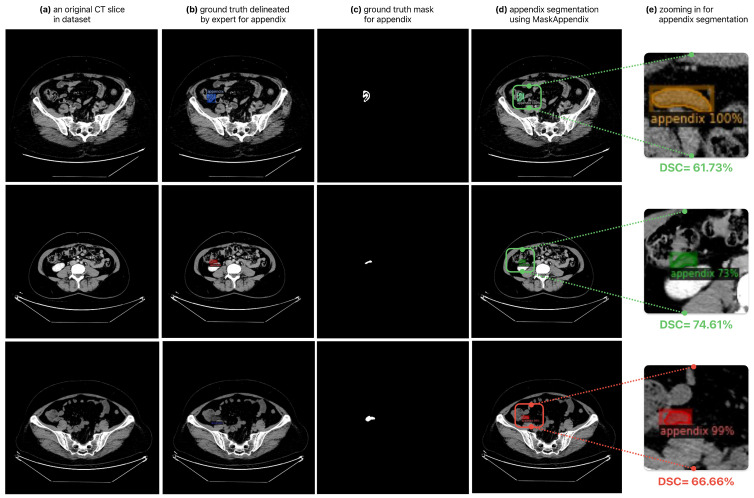
Appendix regions segmented with lower performance using the proposed MaskAppendix model in some CT slices.

Evaluating the results of the experimental studies conducted for the automatic segmentation of the appendix region in the appendix abdomen CT slice dataset created for this study, it can be clearly stated that the segmentation results of the MaskAppendix model (Mask R-CNN with ResNet101) are more successful than the segmentation results obtained using state-of-the-art U-Net and DenseNet architectures. Although it has been verified that the proposed method achieves high segmentation scores with key metric measurements on the slices in the dataset, some appendix regions segmented with lower performance by the proposed MaskAppendix model are shown in Figure 8. Although the segmentation results on the images are reasonable, there may be many different reasons for this and similar problems. The low segmentation results for the appendix may be due to the fact that in some CT slices the appendix region is close to or connected to other areas, the patterns are similar, and the boundaries of the region are similar to those of the neighboring areas. On the other hand, the presence of noise in the relevant images, the interweaving of the textural colors of the appendix region and other organs, the fact that the appendix has more than one part, errors made by the expert in marking, and the number of iterations in the training of the MaskAppendix network, which was insufficient to detect the appendix regions in these images, could also have reduced the segmentation success.

**Figure 9:**
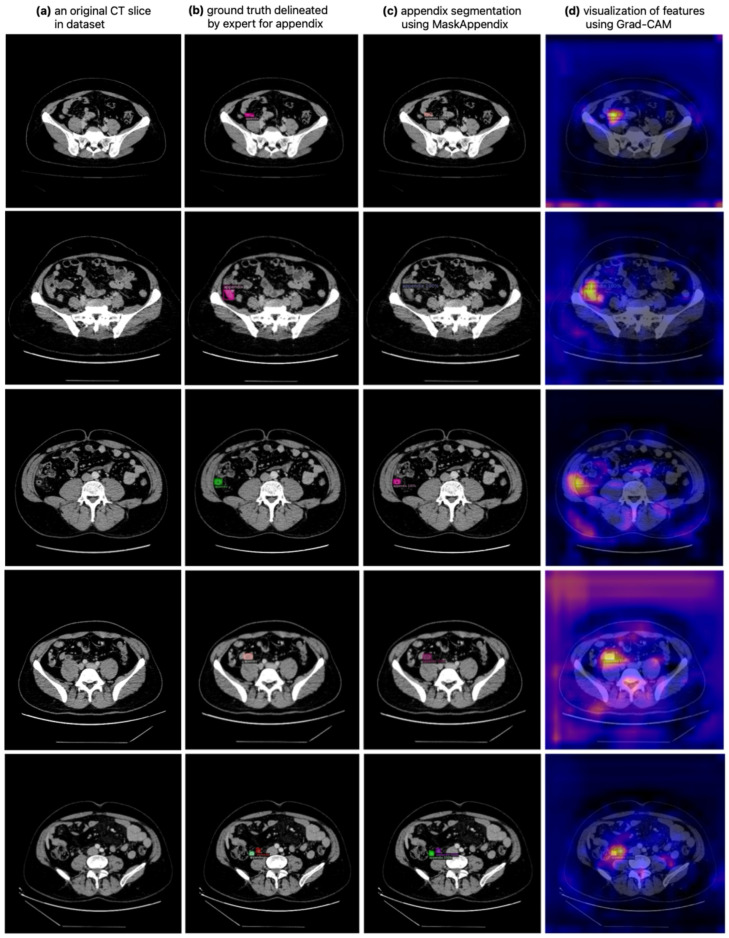
Evaluation of visual results of heatmap feature outputs with Grad-CAM with MaskAppendix segmentation result.

The combination of Mask R-CNN and Grad-CAM improves segmentation accuracy by providing complementary strengths in object detection, feature localization and explainability. Grad-CAM provides a mechanism to highlight the key regions of the image that contribute to predicting a particular class [58]. In the context of appendix segmentation, the combination of Mask R-CNN and Grad-CAM helps Mask R-CNN to focus on the most relevant parts of the image where the appendix is probably located. Rather than being distracted by surrounding anatomical structures, Grad-CAM prompts the model to increase its attention to regions that contribute most to the correct identification of the appendix. In this study, Gradient-weighted Class Activation Mapping (Grad-CAM), a discriminative localization technique, was also used to evaluate whether the proposed MaskAppendix model (Mask R-CNN with ResNet101 backbone) correctly segmented the location of the appendix. Grad-CAM can be applied to any CNN model on images and can produce a more transparent heatmap by generating visual descriptions [59]. In this study, the feature map output from the final convolutional layer of the MaskAppendix model was used to generate Grad-CAM on an input CT slice. Figure 9a shows the expertly delineated ground truth mask of the appendix and its position on an original CT slice is shown in Figure 9b. On the other hand, the automatic segmentation of the appendix in this CT slice with the proposed MaskAppendix model is shown in Figure 9c. Figure 9d shows the Grad-CAM evaluation of the correct segmentation and position of the appendix. It can be seen that the MaskAppendix segmentation result and the visual results of the heatmap features output with Grad-CAM are compatible with each other. By integrating Grad-CAM into the Mask R-CNN framework, the proposed model gained the ability to better understand and highlight the fine details that distinguish the appendix from neighboring anatomical structures. As a result, clinical outcomes can be improved using Grad-CAM as the appendix can be more clearly visualized and diseases such as appendicitis can be diagnosed more quickly and precisely.

Table 6 also evaluates the total runtime of the Mask R-CNN with ResNet50 and Mask R-CNN with ResNet101 (MaskAppendix) models for training and test sets. There is a significant difference in depth between ResNet50 and ResNet101 backbone networks. This is expected to be reflected in the feature training times of the networks. Total time for Mask R-CNN with ResNet50 for training and the time for one Checkpoint Period were 528.6 and 5.29 min, respectively. On the other hand, since the backbone network used for MaskAppendix is deeper, the same times were obtained as 723.6 and 7.24 min, respectively. The test time was calculated as 29.3 s for Mask R-CNN with ResNet50 and 36.5 s for MaskAppendix. As a result, it can be seen that the training and test times obtained according to the depth of the networks used as backbone in the infrastructure of the Mask R-CNN architecture are reasonable and compatible.

In this study, for appendix segmentation, the ground truth masks for the appendix provided by expert physicians in the dataset created with CT scans of healthy individuals and appendicitis patients were important for the generalization of the proposed model. Nevertheless, there are several limitations to the proposed MaskAppendix model, although it shows promising results in appendix segmentation. A larger, more diverse dataset including different imaging conditions would be required to fully assess the robustness and generalizability of the model. In addition, the model has yet to undergo extensive clinical validation, despite performing well in a controlled experimental setting. The impact of the model in real-time clinical settings, including its integration into radiology workflows, remains to be investigated, as does how radiologists interact with its output. In addition, widespread adoption of the proposed model may be challenged by the computational cost of training and deploying the model in real-world environments.

## 5. Conclusions

The contribution of computer-aided tools to assist physicians in the diagnosis of disease from medical images has increased in recent years. It is known that segmentation of these images by manual selection of the RoI by physicians is prone to human failure, requires more time, and consists of too many procedural dependencies. Therefore, automated segmentation of the appendix from CT images can make a significant contribution in terms of both time and cost. This study presents the potential of the proposed MaskAppendix deep learning model on the Detectron2 platform for accurate appendix segmentation from CT images. In the automatic segmentation of the appendix region in CT slices, the results of 87.17% DSC, 78.11% JSI, 21.88% VOE, 0.47 mm ASD, and 3.70 mm HD95 obtained with the proposed approach are more successful than the state-of-the-art U-Net and DenseNet deep learning architectures. Therefore, the obtained results have the potential to improve the accuracy of clinical diagnosis. On the other hand, in the proposed MaskAppendix model, two different networks, ResNet50 and ResNet101, were used as the backbone and their effects on appendix segmentation from CT slices were evaluated by ablation experiments. The ablation studies confirm that higher segmentation performance can be achieved when ResNet101 is used as the backbone in the Mask R-CNN architecture. In addition, the verification of the correct segmentation of the appendix location in the proposed MaskAppendix model was evaluated using a heatmap generated by generating visual annotations on the images using Grad-CAM.

The proposed method can have a significant improvement in the diagnosis of appendicitis and patient care using the MaskAppendix model, which integrates Mask R-CNN with Grad-CAM for automated appendix segmentation. First of all, the precise segmentation provided by the MaskAppendix model can give surgeons a clear understanding of the location, size, and surrounding structures of the appendix. Therefore, the proposed MaskAppendix model can overcome the challenges of accurately and automatically segmenting the appendix on CT scans, allowing radiologists to visualize the appendix with greater clarity. In addition, the method can be particularly valuable in cases where the appendix is not easily distinguishable, such as in patients with abnormal anatomy or conditions. By using Grad-CAM to improve feature localization, the model can identify subtle of appendicitis, improving diagnostic confidence. Moreover, clinicians can provide personalized care based on each patient’s specific anatomy and condition by automating and improving the accuracy of appendix segmentation.

In future studies, the dataset can be expanded by increasing the patient population with CT scans obtained from different resolution, size, and scanner types. In addition, it is planned to improve the existing model and increase the segmentation performance by developing hybrid deep learning architectures and automatic hyperparameter optimization.

## Figures and Tables

**Table 1:** The used CT scanner and some important acquisition parameters.

CT scanner	64-MDCT (multi-slice CT Aquillion 64; Toshiba)
Slice thickness	2.5 mm
Reconstruction interval	0.777 mm
Gantry rotation time	0.6 s
Tube voltage	120 kV
Tube current	200 mA (in average)
The field of view range	From 40 to 50 cm
Image size	512 × 512

**Table 2:** Hardware characteristics of the computer used for experimental studies.

Hardware	Characteristic
Computer	Workstation
Central Processor (CPU)	Intel Core i9-9900 K @ 5 GHz (8 Core/16 Thread)
Memory (RAM) (×2)	16 GB DDR4 2666 MHz
Mainboard	WS Z390 Pro DDR4
GPU (×2)	NVIDIA GeForce GTX 1080Ti 11 GB
Hard disk drive	500 GB SSD + 3 TB SATA 6 Gb 3.5″

**Table 3:** Optimization of the hyperparameters used for the Mask R-CNN architecture run on the Detectron2 platform.

Hyperparameter	Value	Identification
BACKBONE	ResNet50 and ResNet101	Network model for feature extraction and feature mapping
GPU_COUNT	2	Number of GPUs on the computer on which the network runs
NUM_WORKERS	16	The number of worker threads or processes used for data loading
IMS_PER_BATCH	2	The number of images processed in each training batch
BASE_LR	0.001	Learning rate of the network
CHECKPOINT_PERIOD	1000	How often the state of the model is recorded during training
MAX_ITER	100,000	Maximum number of iterations required to complete the training
ANCHOR_GENERATOR.SIZES	(16, 32, 64, 128, 256)	The sizes of the anchors for object detection
NUM_CLASSES	1	Number of classes for CT slices
BATCH_SIZE_PER_IMAGE	256	The number of RoI

**Table 4:** Average results achieved on CT scans in the test set for MaskAppendix and other state-of-the-art methods based on DSC, JSI, VOE, ASD, and HD95 key metrics.

Method	DSC [%]	JSI [%]	VOE [%]	ASD [mm]	HD95 [mm]
DenseNet	79.88	70.61	29.39	1.67	6.89
U-Net	85.94	76.70	23.29	1.24	5.43
MaskAppendix	87.17	78.11	21.89	0.47	3.70

**Table 5:** Comparison of ablation results for ResNet50 and ResNet101 backbone networks in Mask R-CNN deep learning architecture for appendix segmentation from CT scans.

Method	Backbone	DSC [%]	JSI [%]	VOE [%]	ASD [mm]	HD95 [mm]
Mask R-CNN	ResNet50	87.07	77.79	22.21	0.48	2.68
Mask R-CNN	ResNet101	87.17	78.11	21.88	0.47	3.70

**Table 6:** Total runtimes of the Mask R-CNN with ResNet50 and Mask R-CNN with ResNet101 (MaskAppendix) models for training and test sets.

Method	Backbone	Total Time for Training (min)	The Time for a Checkpoint Period (min)	Total Time for Test (s)
Mask R-CNN	ResNet50	528.6	5.29	29.3
Mask R-CNN	ResNet101	723.6	7.24	36.5

## Data Availability

The raw data supporting the conclusions of this article will be made available by the authors on request.
